# Increase of arginase activity in old apolipoprotein-E deficient mice under Western diet associated with changes in neurovascular unit

**DOI:** 10.1186/1742-2094-9-132

**Published:** 2012-06-18

**Authors:** Jérôme Badaut, Jean-Christophe Copin, Andrew M Fukuda, Yvan Gasche, Karl Schaller, Rafaela F da Silva

**Affiliations:** 1 Departments of Pediatrics and Physiology, Linda University School of Medicine, Coleman Pavilion, Room A1120, 11175 Campus Street, Loma Linda, CA, 92354, USA; 2 Division of Neurosurgery, Geneva University Medical Center and Geneva Neuroscience Center, University of Geneva, Geneva, Switzerland; 3 Division of Intensive Care, Geneva University Hospitals and Geneva Neuroscience Center, University of Geneva, Geneva, Switzerland; 4 Institute of Bioengineering, Swiss Federal Institute of Technology Lausanne, Lausanne, Switzerland; 5 Department of Physiology and Biophysics, Federal University of Minas Gerais, Belo Horizonte, Brazil

**Keywords:** Neurovascular unit, Nitric oxide, Arginase, Blood brain barrier, Water channel, Brain aging

## Abstract

Aging and atherosclerosis are well-recognized risk factors for cardiac and neurovascular diseases. The Apolipoprotein E deficient (ApoE−/−) mouse on a high-fat diet is a classical model of atherosclerosis, characterized by the presence of atherosclerotic plaques in extracranial vessels but not in cerebral arteries. Increase in arginase activity was shown to participate in vascular dysfunction in the peripheral arteries of atherosclerotic mice by changing the level of nitric oxide (NO). NO plays a key role in the physiological functions of the neurovascular unit (NVU). However, the regulation of arginase expression and activity in the brain was never investigated in association with changes in the NVU, ApoE deficiency and high fat diet.

Fourteen-month-old ApoE−/− mice on high-fat diet exhibited deposition of lipids in the NVU, impairment of blood–brain barrier properties, astrogliosis and an increase of aquaporin 4 staining. In association with these changes, brain arginase activity was significantly increased in the old ApoE−/− mice as compared to old wild type mice, with an increase in the level of arginase type I in the blood vessels.

In conclusion, aging in this classical mouse model of atherosclerosis induces an increase in the level and activity of arginase I that may impair NO synthesis and contribute to changes in the NVU leading to blood–brain barrier leakage and inflammation.

## Background

Apolipoprotein E deficient (ApoE−/−) mice have been widely used as animal models to study the pathophysiology of atherosclerosis. These mice have the propensity to spontaneously develop atherosclerotic lesions, and ingestion of a Western diet exacerbates the development of plaques in peripheral blood vessels [1]. The model is characterized by a 5 to 10 times increase in serum cholesterol levels and by the local deposition of lipids in the arterial wall. These mice also develop a complex endothelial dysfunction [2], with adhesion of a large number of T cells and macrophages leading to chronic inflammation systemically and also at intraplaques [3,4].

The decrease of nitric oxide (NO) plays a key role in the pathophysiology of atherosclerosis [5,6]. The availability of NO is regulated by very complex mechanisms, which include the availability of L-arginine, the common substrate of nitric oxide synthase (NOS) and arginase. Arginase exists as two genetically distinct isoforms named cytosolic arginase I (ArgI) and mitochondrial arginase II (ArgII) [7]. Increase in arginase expression and activity has been shown to contribute to vascular dysfunction of extracranial blood vessels in aged mice and rats, and also in atherosclerotic mice [8,9]. Clinical observations suggested that cerebral arteries might be more resistant to cholesterol-induced atherosclerosis than extracranial arteries [10]. In the brain of ApoE−/− mice, the deficiency of ApoE was associated with gliosis stimulation, microglia activation [11] and xanthoma accumulation around blood vessels. However, despite these changes, no intravascular plaques were formed [12]. These alterations were postulated to contribute to cognitive dysfunctions [13] due to neuronal and vascular damage. It was shown that blood–brain barrier (BBB) function was altered in ApoE−/− mice [14], with an aggravation of barrier leakage throughout the aging process [15]. While this model has been widely used in the periphery, little is known about the changes in the NVU, which is composed of blood vessels, astrocytes and neurons.

NO plays an important role in the physiological regulation of the nervous system, cerebral blood vessels and cerebral blood flow [16]. Changes in NO/NOS in the brain cortex and hippocampus were associated with cognitive impairments in aged mice and rats [17,18]. In the mouse brain, both arginase enzymes were constitutively expressed in neurons but not in glial cells [19]. A cellular and regional distribution study showed that arginase was highly expressed in the cortex, hippocampus, cerebellum, pons and medulla. Although both ArgI and ArgII are co-expressed in most of the cells studied, the expression of ArgI is more pronounced than ArgII [19]. In aged rats, arginase activity was increased in the frontal cortex and in different sub-regions of the hippocampus [17,18]. Considering that aging and atherosclerosis are risk factors for cardiac and neurovascular diseases, such as stroke, we investigated the regulation of arginase in young mice (1-month-old) and old mice (14-months-old) in a classical model of atherosclerosis, using ApoE−/− mice on a Western diet. We hypothesized that in the murine model of atherosclerosis, brain arginase expression and activity could be altered by aging in association with changes in the NVU properties.

## Material and methods

### Animals and diets

All animal experiments were conducted in accordance with the guidelines of the cantonal veterinary service (Switzerland, Canton de Vaud). The animals were maintained in standard laboratory conditions with a 12/12 h light–dark cycle (lights on at 0700 hours). One- and 14-month-old C57BL/6 J wild type (WT) and ApoE−/− mice (n = 10 for each group) were obtained from Charles River (L'Arbresle Cedex, France). WT mice were fed *ad libitum* on a normal chow diet, whereas ApoE−/− were fed on a Western-type diet containing 15% (w/w) cocoa butter and 0.25% (w/w) cholesterol (Diet W; Hope Farms b.v., Woerden, The Netherlands). At the end of the study protocol, blood was sampled, the animals were euthanized, the brains were isolated, snap frozen on dry ice, and stored at −80 °C until further processing.

The anterior brain was cut in 50 μm coronal sections. Cerebral cortical and striatal tissues were isolated from the first 20 coronal sections (1 mm in total), then pooled and used for protein extraction. The rest of the brain was serially cut in 10 μm coronal sections, attached to super frost slides, air-dried and kept at −80 °C until further processing. Before staining, brain sections were fixed in 100% cold acetone for 10 minutes. The group of ApoE−/− mice on the Western-type diet will be referred to in the rest of the Material and methods and the Results section as ApoE−/−.

### Plasma lipid analysis

Mice were starved for 12 hours and blood was collected into heparin-coated tubes by cardiac puncture. Plasma was obtained by centrifugation of the blood for 15 minutes at 4,500 rpm at 4 °C and stored at −80 °C until further processing. Total cholesterol, low density lipoprotein (LDL) and high density lipoprotein (HDL) levels were determined using an automated clinical chemistry analyzer.

### Arginase activity

Protein lysate was extracted with arginase buffer as previously described [20]. One hundred micrograms of protein were used for arginase activity assay and the rest for Western blot analysis. Arginase activity in frontal sections was measured by colorimetric determination of urea formation according to a previously published procedure [21].

### Western blot analysis

Ten, 25 or 50 μg of total protein lysate were electrophoresed and transferred to nitrocellulose membranes (GE Healthcare Biosciences, Pittsburgh, PA, USA). Membranes were incubated overnight at 4 °C with the following primary antibodies: mouse anti-ArgI (1:500, BD Biosciences, Allschwil, Switzerland), rabbit anti-ArgII (1:200, Santa Cruz Antibody, Santa Cruz, CA, USA), and mouse anti-β-actin (1:10,000, GE Healthcare Biosciences, Pittsburgh, PA, USA). After several washes and blocking with 5% milk, membranes were incubated with either goat anti-rabbit or goat anti-mouse IgG horseradish peroxidase-linked secondary antibodies (1:1,000, Amersham). Immunoreactivity was detected by enhanced chemiluminescence (Amersham). Protein expression was quantified using a Kodak Image Station 2000R and with Kodak 1D Image Analysis Software (Kodak) . ArgI and ArgII protein expression levels were calculated as their ratio to β-actin housekeeping protein.

### Lipid deposition analysis

Cortical lipid deposition was determined by classical Sudan IV staining followed by hematoxylin staining [22] and later analyzed by microscopy.

### Immunohistochemistry

After cold acetone fixation, brain sections were treated in 0.1% Triton X-100 in PBS for five minutes, then incubated overnight at 4 °C with the following primary antibodies diluted in PBS containing 5% normal goat serum: rabbit anti-ArgI (1:100, Santa Cruz Antibody), rat anti-laminin (1:500, BD Biosciences), rabbit anti-aquaporin 4 (anti-AQP4, 1:400, Chemicon, Temecula, CA, USA), mouse anti-Claudin 5 (1:200, Invitrogen, Grand Island, NY, USA), rabbit anti-GFAP (1:500, Chemicon), and mouse anti-neuN (1:500, BD Biosciences). After several washes in PBS (five minutes each), sections were incubated with the appropriate secondary antibody: alexa540-conjugated goat anti-rabbit IgG, alexa488-conjugated goat anti-rat IgG, alexa488-conjugated goat anti-mouse IgG (1:500, BD Biosciences) and infrared-dye680-conjugated goat anti-mouse IgG (1:1,000, Rockland, Gilbertsville, PA, USA) for 45 to 60 minutes at room temperature. Immunofluorescent sections were examined on a Zeiss Axiovert 135 microscope (Feldbach, AG, Switzerland) or with the Odyssey infrared scanner (LI-COR, Lincoln, NE, USA). Digital images were acquired in the same conditions to allow comparative analysis of fluorescence intensity, blood vessels and neuronal counting.

Aquaporin 4 (AQP4) and GFAP immunoreactivities were quantified in three different cortical fields (422 μm × 338 μm) in WT and ApoE−/− mice as previously published [23]. Optical density (OD) was measured by a blinded experimenter using the morpho-expert software on the non-treated images acquired using the Zeiss Axiovert (Explora-Nova, La Rochelle, France). NeuN and laminin immunostaining were quantified in three cortical fields (422 μm × 338 μm) respectively by counting the number of NeuN positive cells and by measuring the surface of the laminin staining with the Mercator software (Explora-Nova, France, [24]). Claudin 5 immunoreactivity was quantified using an Odyssey infrared scanner and converted into average integrated intensities [25].

### IgG staining for blood brain barrier (BBB) evaluation

BBB integrity was assessed by measuring the level of IgG extravasion into the brain. Coronal brain sections were incubated for four hours at room temperature with an infrared-dye 800-conjugated goat anti-mouse IgG antibody (1:500, Rockland) diluted in PBS containing 0.1% Triton X-100 and 1% bovine serum albumin. After washing, fluorescent intensity was quantified using an Odyssey infrared scanner and converted into average integrated intensities [25].

### Statistics

Data were analyzed using one-way ANOVA and Bonferroni *post hoc* test or unpaired *t* test (Graph Pad Prism 5.0; GraphPad Software Inc., San Diego, CA, USA). Data are presented as mean ± SEM and *P*-values ≤0.05 were considered significant.

## Results

### Plasma concentration of cholesterol, HDL, and LDL in young and old WT and ApoE−/− mice

**Figure 1:**
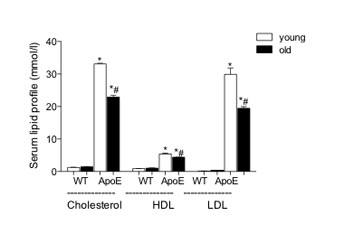
**Quantification of plasma cholesterol, HDL and LDL levels.** Each column represents mean ± SEM (n = 5 per group). * *P* <0.05 as compared to age-matched WT mice and # *P* <0.05 as compared to young mice of the same genotype.

As stated in the introduction, the ApoE−/− mouse on a Western diet is a well-characterized model of atherosclerosis with high blood levels of cholesterol, HDL and LDL contributing to the formation of plaques in peripheral blood vessels (data not shown). Here, plasma concentrations of cholesterol, HDL and LDL were significantly increased in young and old ApoE−/− mice as compared to age-matched WT mice (Figure 1). Old ApoE−/− mice on a Western diet had statistically reduced concentrations of cholesterol, LDL and HDL (22.88 ± 0.60 mmol/l, 19.44 ± 0.55 mmol/l and 4.40 ± 0.48 mmol/l respectively) as compared to young ApoE−/− mice fed for four weeks on the same diet (33.11 ± 0.22 mmol/l, 29.90 ± 1.95 mmol/l, 5.36 ± 1.69 mmol/l respectively, Figure 1). There was no change in plasma concentrations of cholesterol, HDL and LDL between young and old WT mice.

### Lipid deposition

**Figure 2:**
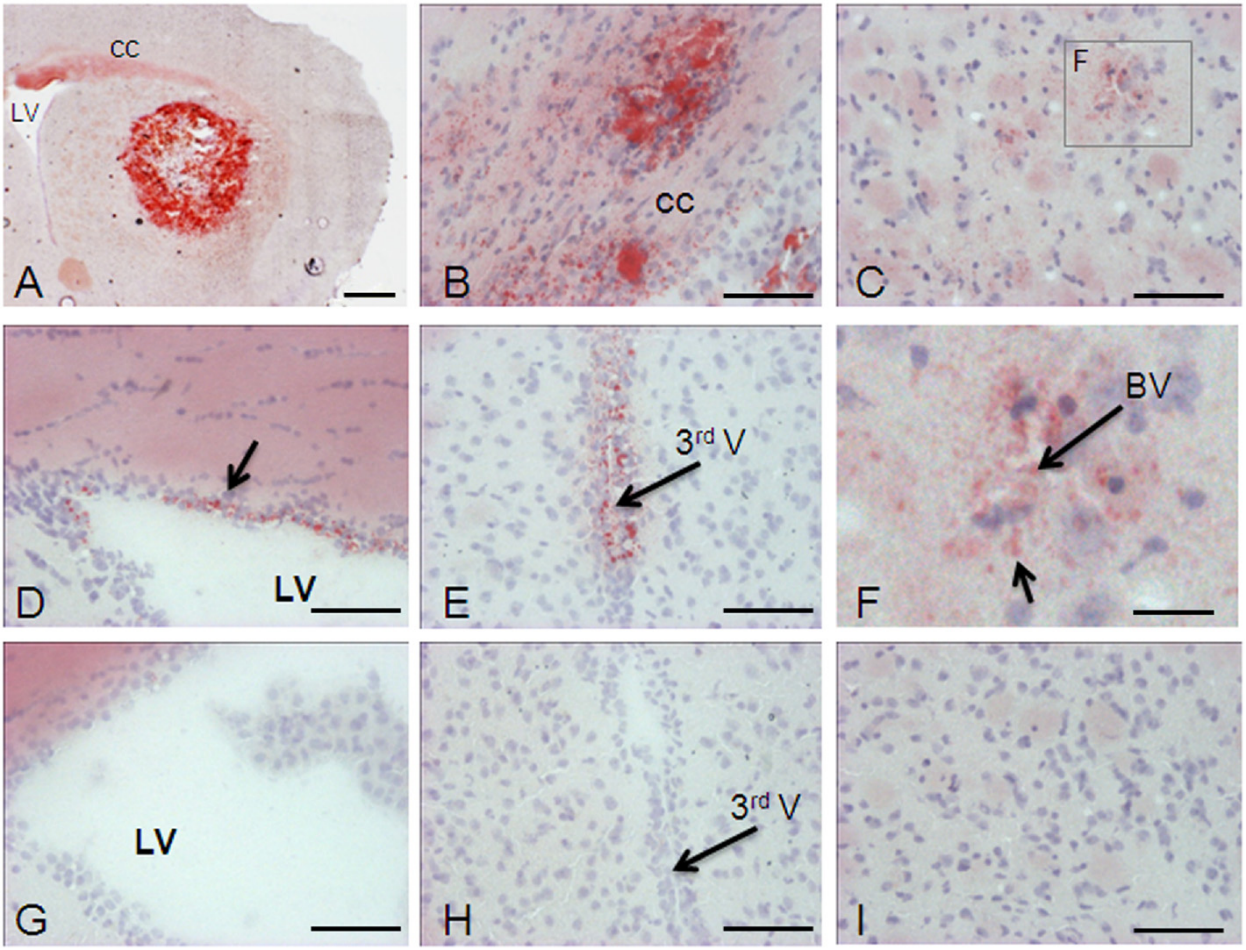
**Lipid deposition in the brain of old ApoE−/− mice.** Representative photomicrographs of lipid deposition in the brain of old ApoE−/− mice (**A to F**) as compared to age-matched WT mice (**G** to **I**, n = 9 per group). Lipid depositions were observed in the perivascular space adjacent to the lateral ventricles and third ventricle, and in the corpus callosum. **A**: striatum; **B**: corpus callosum (CC); **C** and **I**: cortex; **D** and **G**: lateral ventricle (LV); **E** and **H**: third ventricle (3rd V); **F**: brain vessels (BV). Scale bar = 500 μm in **A**, 100 μm in **B** to **E** and **G** to **I**, and 30 μm in **F**.

Brain coronal sections of old ApoE−/− mice showed positive staining for Sudan IV oil red (Figure 2A to 2F). Lipid droplets were found in the corpus callosum (Figure 2B), subventricular zones (Figure 2D, E), and around blood vessels in the cortical area (Figure 2C, F). In one old ApoE−/−, an intense and large Sudan IV staining was observed in the left striatum suggesting an accumulation of lipid droplets (Figure 2A). No cerebral lipid depositions were observed in old WT mice (Figure 2G to 2I) nor in young ApoE−/− or WT mice (data not shown).

### Neurovascular alterations in the cortex of WT and ApoE−/− mice

Accumulation of lipid droplets in close contact with cerebral blood vessels in ApoE−/− mice prompted us to investigate changes in BBB properties and in the expressions of GFAP and AQP4.

**Figure 3:**
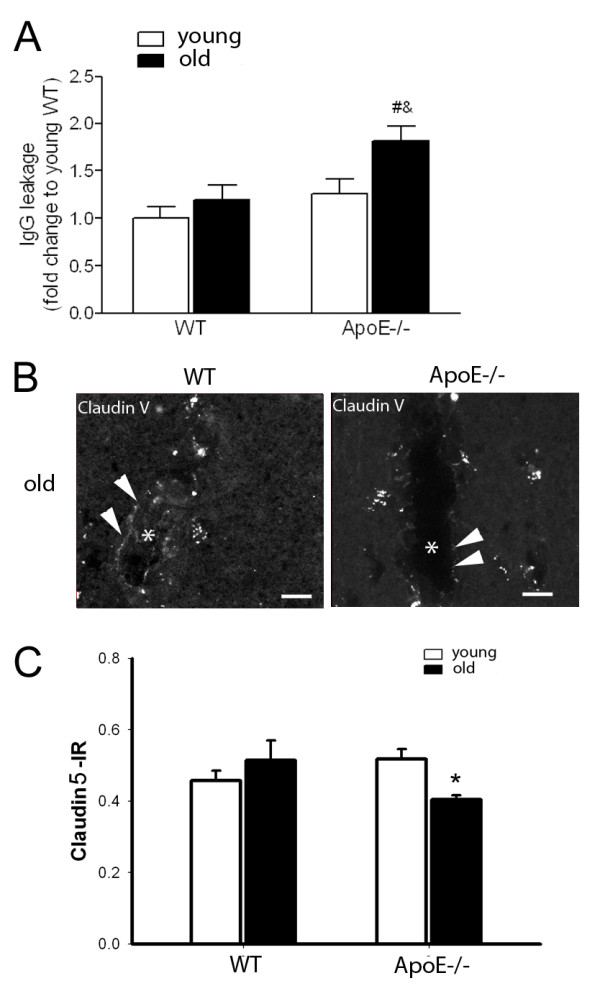
**BBB changes in cerebral cortex of WT and ApoE−/− mice. A**) Age dependent blood brain barrier impairment in ApoE−/− mice. Quantification of IgG leakage in brain sections stained with anti-IgG antibody (n = 7 to 9 per group). Each column represents mean ± SEM. # *P* <0.05 young ApoE−/− mice vs old ApoE−/− mice and *P* <0.05 old WT mice vs old ApoE−/− mice. **B**) Claudin 5 staining (arrow heads) along the cortical blood vessels in the old WT and ApoE−/− mice. The lumen is marked with a star. Claudin 5 staining is decreased in old ApoE−/− mice. **C**) Quantification of the intensity of the Claudin 5 immunoreactivity (IR) in cortical layers of young and old WT and ApoE−/− mice. Each column represents mean ± SEM (n = 4 per group). * *P* <0.05 young ApoE−/− mice vs old ApoE−/− mice.

The intensity of IgG staining in the brain, used as a marker of BBB disruption, was significantly higher in old ApoE−/− mice compared to age-matched WT mice (Figure 3A). In young mice, IgG staining was slightly but not significantly increased in ApoE−/− mice as compared to age-matched WT mice. IgG staining was also significantly higher in old ApoE−/− mice as compared to young ApoE−/− mice.

Paralleling the increase of IgG extravasation, a decrease in Claudin 5 staining was observed in the cortical blood vessels of old ApoE−/− mice compared to WT mice (Figure 3B). Quantification analysis showed that aging in ApoE −/− mice significantly decreased Claudin 5 immunoreactivity compared to young ApoE−/−mice (0.51 ± 0.05 A.U., 0.40 ± 0.01 A.U, respectively, Figure 3C).

**Figure 4:**
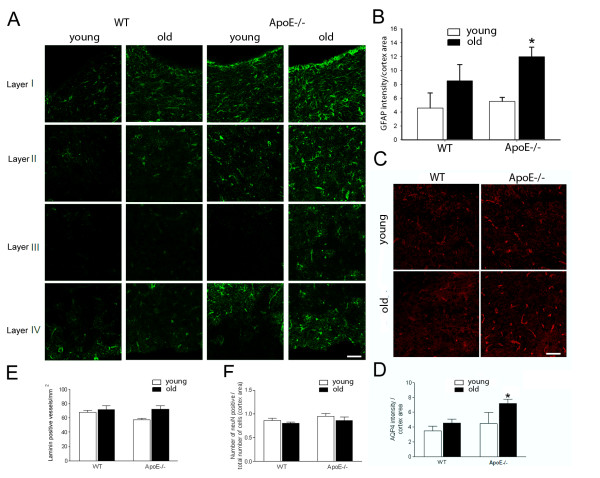
**Neurovascular alterations in cerebral cortex of WT and ApoE−/− mice. A**) GFAP staining in the different brain cortical layers in young and old WT and ApoE−/− mice. Immunohistochemistry for GFAP is distributed in a gradient in cortical cross-sections of young and old WT and ApoE−/− mice as previously described. GFAP staining was increased with aging regardless of the mice strain. The increase is higher in the old ApoE−/− mice, with presence of GFAP positive astrocytes throughout all of the cortical layers. Scale bar = 50 μm. **B**) Quantification of the intensity of the GFAP staining in cortical layers of young and old WT and ApoE−/− mice. Each column represents mean ± SEM (n = 4 per group). * *P* <0.05 young WT mice vs old ApoE−/− mice and old WT mice vs old ApoE−/− mice. **C**) AQP4 immunohistochemistry in the cortex of the young and old WT and ApoE−/− mice. AQP4 expression is not increased in old WT. In contrast, in ApoE−/−, the AQP4 labeling is increased in the astrocyte endfeet in contact with the blood vessels. Bar = 40 μm. **D**) Quantification of the intensity of the AQP4 staining in cortex cross-section of young and old WT and ApoE−/− mice. Each column represents mean ± SEM (n = 4 per group). * *P* <0.05 ApoE−/− young vs ApoE−/− old. **E**) Counting of the number of positive laminin staining did not show any significant differences between the WT and ApoE−/− mice and between young and old (n = 6 per group). **F**) Counting of the number of positive NeuN cells did not show any significant difference between the WT and ApoE−/− mice and between young and old (n = 6 per group).

Similar to the changes observed in BBB permeability, an increase in GFAP expression in the cortex of ApoE−/− mice was seen with aging (Figure 4A). In WT mice, GFAP staining was observed in young animals in cortical layers I and IV but was also distributed in layer II in old animals. In young ApoE−/− mice, GFAP staining was observed in cortical layers I, II and IV. In old ApoE−/− mice, GFAP staining was very pronounced and observed in layers I, II IV and, additionally, in layer III. Quantification of GFAP staining in cortical layers showed a trend of higher expression of GFAP in old ApoE−/− mice when compared to young ApoE−/− mice (12.0 ± 0.05 A.U. vs. 8.51 ± 2.35 A.U., respectively) and a significant increase of GFAP when compared to young and old WT mice (12.0 ± 0.05 A.U. vs. 4.60 ± 2.17 A.U., 12.0 ± 0.05 A.U. vs. 5.54 ± 0.58 A.U, respectively, Figure 4B)

AQP4, a water channel present in astrocyte endfeet, was present in close contact to the blood vessels (Figure 4C). AQP4 staining was increased in the young ApoE−/− mice group as compared to the young WT mice, although it did not reach significant levels (Figure 4D). However, aging significantly increased the expression of AQP4 in the ApoE−/−mice, but not in the WT mice (Figure 4D).

The increase of GFAP and AQP4 in old ApoE−/− mice did not coincide with an increase in vascular density, as assessed by quantification of laminin density in the different groups that showed no differences (Figure 4E). Moreover, no difference in neuronal density was detected between the different groups, suggesting identical neuronal viability among the different groups (Figure 4F).

### Changes in arginase activity and expression with aging

**Figure 5:**
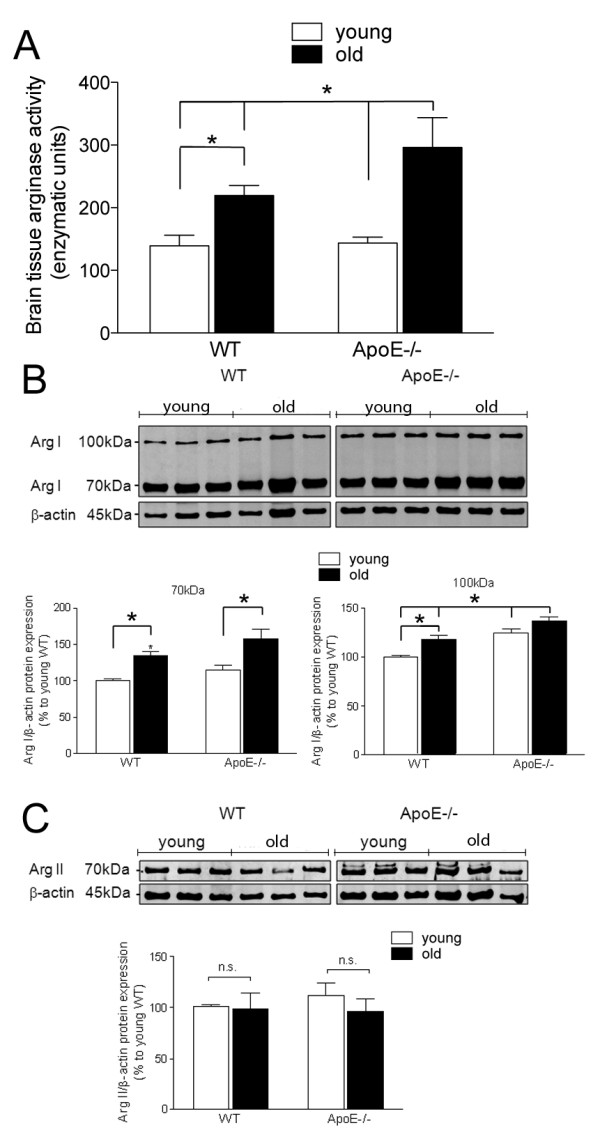
**Arginase activity and expression in the brain of young and old WT and ApoE−/− mice. A**) The highest level of arginase activity was found in old ApoE−/− mice. Each column represents mean ± SEM (n = 10 per group). * *P* <0.05 young WT vs old WT, young ApoE−/− vs old ApoE−/−, old ApoE−/− vs young WT, and old ApoE−/−vs old WT. **B**) Representative Western blots of ArgI (100 and 70 kDa bands) and quantification. Both bands of ArgI protein were significantly increased with aging in WT and ApoE−/− mice. In addition, the ArgI 100 kDa band was significantly up-regulated in ApoE−/− mice as compared to both age-matched WT. Each column represents mean ± SEM (n = 6 per group). * *P* <0.05 young WT vs old WT, young ApoE−/− vs old ApoE−/−, young WT vs young ApoE−/−, old ApoE−/− vs young WT, and old ApoE−/− vs old WT. **C**) Representative Western blots of ArgII (70 kDa band) and quantification. No significant changes in ArgII level were observed between WT and ApoE−/− mice (n = 6 per group).

Arginase activity was significantly increased in the brain of old WT mice (219.51 ± 15.98 μ/mg of protein) as compared to young WT mice (139.08 ± 17.04 μ/mg of protein) (Figure 5A), suggesting that aging altered arginase activity. Similarly, arginase activity was higher in old ApoE−/− mice (296.24 ± 47.51 μ/mg of protein) as compared to young ApoE−/− mice (143.56 ± 9.50 μ/mg of protein; Figure 5A). Arginase activity in old ApoE−/− mice was also significantly higher than in old WT mice (Figure 5A).

The level of arginase I and II were investigated in brain sections of WT and ApoE−/− mice to assess whether the enzymes were regulated by the aging process. Both WT and ApoE−/− mice showed two distinct forms of ArgI, with apparent molecular weights of 70 and 100 kDa (Figure 5B). Both the 70 and 100 kDa forms were significantly increased with age in WT mice by 33% for the 70 kDa form and by 18% for the100 kDa (Figure 5B). In the ApoE−/− mice, aging increased ArgI by 42% for the 70 kDa form and by 13% for the 100 kDa form (Figure 5B). Brain tissues of young and old ApoE−/− mice had markedly higher levels of the 100 kDa form as compared to age-matched WT animals (24% higher in young mice and 18% higher in old mice; Figure 5B).

A 70 kDa form of ArgII was detectable by Western blot in both WT and ApoE−/− brain extracts (Figure 5C). No significant changes in ArgII expression were observed between WT and ApoE−/− mice and aging did not alter ArgII expression (Figure 5C).

**Figure 6:**
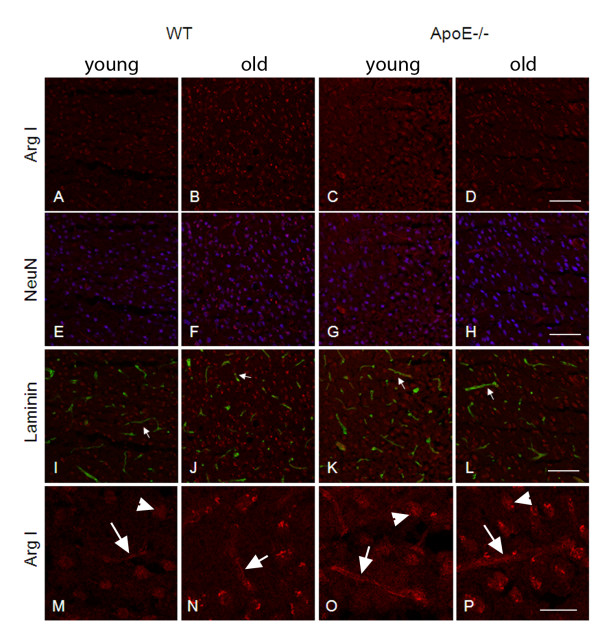
**ArgI localization in the cerebral cortex of WT and ApoE−/− mice.** Representative images of ArgI staining (in red) in frontal cerebral cortex from young (**A, E, I, M**) and old (**B, F, J, N**) WT mice and from young (**C, G, K, O**) and old (**D, H, L, P**) ApoE−/− mice (n = 6 per group). Single staining is shown in the first (A-D) and last row (M-P). Arrows show a vascular localization. Arrowheads show a nuclear localization in extravascular cells. Double staining with NeuN (in blue) and laminin (in green) are shown on the second and third row, respectively. Scale bar = 100 μm in A to L and 40 μm in M to P.

Cellular localization of ArgI was assessed by immunostaining in the cortex of the four groups of mice. ArgI staining was observed in the nuclei of extravascular brain cells but also in the cerebral capillaries in all four groups (Figure 6A to 6D and Figure 6M to 6P). Staining intensity was increased in old WT and ApoE−/− mice and in young ApoE−/− mice as compared to young WT mice, in accordance with the Western blot results. Double staining demonstrated a co-localization of ArgI with NeuN (Figure 6E to 6H), a neuronal marker, and ArgI with laminin (Figure 6I to 6L), a marker of blood vessels, suggesting cerebral expression of ArgI in neurons and blood vessels.

## Discussion

Atherosclerosis is a risk factor for brain ischemia and the incidence of stroke increases with aging. Interestingly, cerebral arteries are known to be more resistant to cholesterol-induced atherosclerosis than arteries in the periphery [26]. The ApoE−/− mouse on a Western diet is a well-established model of atherosclerosis. Rather than formation of plaques as observed in extracranial blood vessels, accumulation of droplets was observed in the border of the ventricles and around blood vessels in the brain parenchyma of old ApoE−/− mice. Lipid deposition was associated with changes in the NVU: a compromised BBB and an increase in the level of perivascular AQP4 and GFAP in the brain cortex. Similar to what was observed in peripheral blood vessels, arginase activity was significantly increased in the brain of ApoE−/− mice with aging. Between the two Arg isoforms, only ArgI expression was altered with aging, with a pronounced increase of expression in the cortical blood vessels.

NO plays a key role in the physiological regulation of the nervous system, cerebral blood vessels and also in extracranial blood vessels. Previous studies have shown that endothelial and inducible NOS (eNOS and iNOS respectively) play a role in the development of atherosclerotic plaques in the aorta of ApoE−/− mice [27,28]. However, the mechanism of NO regulation is complex and NOS is not the exclusive mediator. Arginase competes with NOS for the common substrate, L-arginine, indirectly modulating the level of NO formation. Certain pathological states, such as atherosclerosis, diabetes and hypertension, can differentially modulate the expression and activity of arginase in vascular cells. In our model, aortic arginase activity is markedly increased in atherosclerosis using ApoE−/− mice on a Western diet as compared to WT mice [21]. Recently, Ryoo and colleagues described endothelial ArgII as a novel molecular target for the treatment of atherosclerosis [29]. They showed that an increase in vascular arginase activity contributes to the mechanism of endothelial dysfunction and plaque development in the aorta of ApoE−/− mice [29]. The activation of the arginase isoforms seems to be tissue-specific and, so far, little is known about the changes in the NVU in ApoE−/− mice on a Western diet with aging.

In the old ApoE−/− mice, the NVU microenvironment is significantly altered by deposition of lipid droplets that might increase the extracellular osmotic pressure. The presence of these droplets may contribute to the leakage of the BBB by a decrease of Claudin 5 expression, consequently resulting in a higher level of extravascular IgG. These changes are paralleled by an increase of GFAP and AQP4 expression. Old ApoE−/− mice with an elevated expression of AQP4 subjected to stroke might have a worse outcome with a higher degree of edema formation as previously proposed with AQP4 overexpression in transgenic mice [30]. These changes in the properties of the NVU could contribute to a worse outcome after stroke in aged patient with atherosclerosis.

Based on our knowledge of the extracranial blood vessels, we hypothesized that the changes in the NVU are partly due to the increase of arginase activity, which in turn, could affect the availability of NO. Our results showed a moderate increase in cerebral arginase activity in old WT mice. These results are consistent with two previous works demonstrating that normal aging affects arginase activity in rat brain [17,18]. The combination of ApoE deletion and high-fat diet exacerbated the activation of arginase with aging. Our observation of 70 kDa forms of ArgI and II in the brain, instead of 38 kDa forms like in the liver and kidney, is consistent with the literature [17,18,31]. It was supposed that Arg could form dimers under certain experimental conditions. In addition, we found a specific 100 kDa form of ArgI, which has not been characterized yet. Natural aging induced a significant increase in ArgI expression, but not in ArgII expression. This increase was further exacerbated in our murine atherosclerosis model. Our results are in contrast to previous rat studies, which revealed a unique change in arginase activity, but not in protein expression [17,18,31]. These differences could be explained by inter-species specificity.

**Figure 7:**
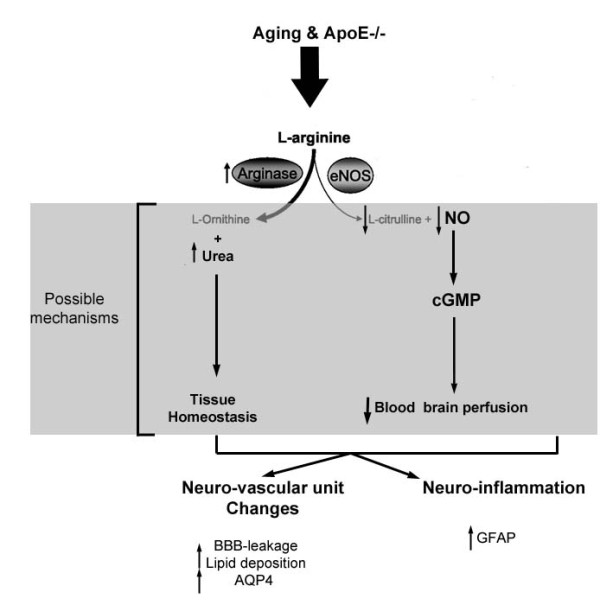
**Diagram of the potential molecular mechanisms.** The combination of aging and ApoE deficiency is associated with an increase of arginase activity with no changes in eNOS. Higher arginase activity would lead to a decrease of NO availability caused by a depletion of L-arginine. One of the possible consequences would be a decrease in blood brain perfusion and facilitated lipid deposition around the microvessels. The increase of arginase activity could also contribute to an increase of urea and induce changes in the NVU homeostasis. The changes in the NVU and neuroinflammation, characterized by the opening of the BBB and increase of GFAP and AQP4 expression, might be a consequence of the decrease of NO, decrease of blood brain perfusion, and increase of urea.

In the present work we show that ArgI expression is increased in neurons and more accentuated in blood vessels in the cortex of old atherosclerotic mice. In the peripheral vasculature, arginase activity was shown to be increased, leading to a decrease in NO and an accumulation of radical oxygen species [32]. It is still unclear how ArgI is involved in the aging process of ApoE −/− mice brain. However, we speculate that the increase in arginase activity would lead to a decrease of NO availability, resulting in a decrease of blood brain perfusion, which in turn could facilitate lipid deposition around the microvessels and account for the changes in astrocyte proteins (for details, see Figure 7). Law and colleagues showed that the aging process increases NOS activity in mice brain cortices. However, ApoE deficiency had no further effects on total NOS activity in both young and aged groups [33]. The methodology used in their study did not distinguish the contribution of the distinct NOS isoforms nor measured the direct NO production *in situ*. Arginase can alter NO formation not only by regulation of the enzymatic function of NOS, but also by modulating the formation of peroxinitrite, dimerization, phosphorylation of eNOS, and changing eNOS and iNOS expression. Therefore, a more detailed investigation would be necessary to clarify the implication of arginase increase in our model.

## Conclusions

The present work showed for the first time that arginase isoforms are differentially regulated in the brain of aging WT and ApoE−/− mice. The combination of ApoE deletion and high-fat diet accentuated the increase of ArgI expression and activity in correlation with changes in NVU, such as BBB impairment and an increase of gliosis. These results suggest further investigation on the role of arginase as an alternative modulator of NO in the brain during aging.

## Abbreviations

ApoE−/−, Apolipoprotein E deficient mice; AQP4, Aquaporin 4; ArgI, Arginase I; ArgII, arginase II; BBB, Blood–brain barrier; CC, corpus callosum; GFAP, Glial fibrillary acidic protein; HDL, High density lipoprotein; LDL, Low density lipoprotein; LV, lateral ventricle; NeuN, Neuronal nuclei; NO, Nitric oxide; NOS, nitric oxide synthase; NVU, Neurovascular unit; OD, Optical density; WT, Wild type.

## Competing interests

The authors declare that they have no competing interests.

## Authors’ contributions

JB and RFS designed the experiments, analyzed data and wrote much of the manuscript. JCC and AF helped in the experiments and the editing of the manuscript. KS and YG helped in the editing of the manuscript. All authors have read and approved the final version of this manuscript.
